# The Price of Preparing for War

**DOI:** 10.1289/ehp.112-a1004

**Published:** 2004-12

**Authors:** Charles W. Schmidt

Located a few miles from Anchorage, Alaska’s Eagle River Flats is a coastal saltwater marsh teeming with fish, wildlife—and unexploded mortar and artillery shells. The marsh lies on the Department of Defense’s (DOD) 62,000-acre training facility at Fort Richardson, headquarters to the Army’s Alaskan command and control units. Since World War II, Eagle River Flats has been Fort Richardson’s primary “ordnance impact zone,” where soldiers stationed at the fort come to train with live munitions.

Environmental assessments undertaken at the Flats by the Army have revealed high levels of contaminants including heavy metals, explosive compounds, and white phosphorus, a toxic agent used to generate smoke cover on the battlefield. It was this contamination with white phosphorus, which can damage bones and major internal organs, that in 1994 landed Eagle River Flats on the Superfund National Priorities List, a U.S. Environmental Protection Agency (EPA) compilation of the nation’s most polluted properties. Since then, the Army has been conducting an EPA-approved effort to clean up the white phosphorus.

But in April 2002 the DOD was sued by a citizens’ coalition urging the Army to address remaining contamination problems at the Flats. Among the plaintiffs were the indigenous Chickaloon Indians, who claimed the Army’s use of live munitions was polluting traditional hunting and fishing grounds. The suit also charged that unexploded mortar rounds and artillery shells in the area were leaching toxic chemicals that were migrating to nearby Cook Inlet. The plaintiffs’ attorney, Scott Allen of the San Francisco, California–based law firm Cox and Moyer, says the suit requested that the Army remove some 10,000 unexploded mortar rounds and artillery shells from the area (the number estimated in the Army’s 1998 proposed Superfund cleanup plan), remediate toxic contamination, and abstain from using the range for bombing exercises until a Clean Water Act permit had been obtained for munitions discharges.

When confronted with the lawsuit, the DOD took its case to Congress. There, it argued that the laws on the books were not intended to be applied to operational military ranges in this way, citing long-standing past state and federal regulatory interpretation and practice. The DOD further argued that suits like those brought at Eagle River Flats, if successful, could set a legal precedent whereby environmental litigants could halt military training and thus undermine troop readiness on the battlefield.

Before the 2002 lawsuit even arose, the DOD had proposed new legislation called the Readiness and Range Preservation Initiative (RRPI) to prevent just such lawsuits attempting to use hazardous waste laws to limit training. The RRPI calls for exemptions from a number of environmental laws on more than 8,000 operational DOD training ranges, a land area equal to roughly 24 million acres. Under this proposed new legislation, munitions would not be subject to hazardous waste permitting or cleanup requirements as long as they remain on operational ranges.

## Military Readiness and Pollution

Preparing for war is a heavily industrialized mission that generates fuel spills, hazardous waste, and air pollution. The DOD owns more than 10% of the 1,240 sites currently on the National Priorities List, and has estimated the cost of cleaning up these sites at approximately $9.7 billion. In addition to lead and a variety of solvents, training facilities release munitions constituents including perchlorate (a thyroid toxicant), RDX (an explosive compound and neurotoxicant), and TNT (an explosive compound linked to anemia and altered liver function).

Nearly 1 in 10 Americans live within 10 miles of a DOD Superfund site—a sometimes perilous proximity. The Massachusetts Military Reservation, for instance, a 34-square-mile multi-use training facility in Cape Cod, is slowly leaching solvents, jet fuel, RDX, and perchlorate into the area’s sole aquifer, a drinking water source for up to 500,000 people at the height of tourist season.

Military aircraft from DOD facilities also generate noise and air pollution. For instance, in 1996, the most recent year for which data are available, more than 50,000 military flights contributed to the heavy air traffic over Washington, D.C. According to the Democratic Committee on Energy and Commerce, these flights emitted 75 tons of nitrogen oxides and volatile organic compounds, which generate smog. In 1999, the Sierra Army Depot, located 55 miles northeast of Reno, was California’s leading air polluter, according to the EPA Toxics Release Inventory. The base released some 5.4 million pounds of toxic chemicals that year, including aluminum, copper, and zinc fumes.

As of this publication, Congress has approved legislation requested by the DOD amending the Migratory Bird Protection Act, portions of the Endangered Species Act, and the Marine Mammal Protection Act. Now, the DOD is seeking changes through the RRPI to certain hazardous waste laws—specifically, the Comprehensive Environmental Response, Compensation, and Liability Act (CER-CLA), the Resource Conservation and Recovery Act (RCRA), and the Clean Air Act (CAA). The DOD acknowledges that these laws have never been shown to have interfered with specific military training, but says it can’t afford to wait until training is shut down before it acts. As evidence of the need to act now, the DOD points to a number of lawsuits and “close calls,” including the case at Eagle River Flats and the Navy’s 2002 temporary closure of its Farallon de Medinilla live-fire training range in the Pacific. That closure followed a lawsuit filed by the Center for Biological Diversity alleging that bombing at the range was killing protected migratory birds.

The DOD argues that even the threat of interference by hazardous waste litigation justifies its aims. Joe Willging, an environmental lawyer with the DOD General Counsel’s office, says in reference to the Farallon de Medinilla closure, “We don’t feel it’s wise to wait for that kind of train wreck to see if we are going to lose in litigation. . . . Our job is to send soldiers, sailors, airmen, and Marines into combat environments in the absolute best-prepared way we can. You can’t do that if you introduce artificialities into training. We want to maintain the ability to use those ranges in the optimum way based on military readiness considerations, not on other considerations.”

## Questions of Scope

Top environmental officials in nearly every state oppose the RRPI, as do 39 state attorneys general. Their opposition is based on the DOD’s historic environmental record and growing reputation among state officials for routinely shirking its environmental responsibility. “The DOD has a consistent track record in litigation going back decades for trying to get out of its environmental requirements,” says Daniel Miller, Colorado’s assistant attorney general for environment. (DOD officials claim, however, that the department’s current compliance with environmental requirements is comparable to that of private industry in almost all environmental programs.)

The main goal of the RRPI is to ensure that both munitions and their constituents are exempt from CERCLA and RCRA hazardous waste classifications as long as they remain on operational ranges. Once the range closes or if the munitions or their constituents migrate offsite or pose an “imminent and substantial danger” to human health or agriculture, then CER-CLA and RCRA authority would come into force. At that point, according to the DOD, the relevant environmental agencies would assume jurisdictional authority and impose monitoring requirements and cleanup orders to address the offsite migration at the contamination’s source.

Finally, the RRPI seeks a three-year extension in the DOD’s obligation to demonstrate compliance with state plans to meet CAA standards for ozone, carbon monoxide, and particulate matter. The DOD claims the extension would provide flexibility in its decisions about where to field and base new weapons and aircraft, noting that military emissions typically are less than 0.5% of state emission quotas.

However, state attorneys general disagree with the DOD’s reading, and have expressed concern that the RRPI would effectively mean states could not require the DOD to take any action to address munitions-related contamination on a range—even if that contamination were to migrate offsite and contaminate drinking water supplies—unless regulators could prove imminent and substantial endangerment from the contamination. Further, says Steve Taylor, a national organizer with the Military Toxics Project, an environmental group based in Lewiston, Maine, without their normal authority to order sampling when warranted either offsite or at the source of contamination, regulators cannot possibly demonstrate the imminent and substantial endangerment required to invoke their emergency powers.

Thus, critics argue, the DOD assumes exclusive control over its facilities, assuming an inappropriate level of oversight given the department’s history with environmental compliance. The problem with this approach, Taylor emphasizes, is that munitions contamination that spreads offsite is likely to be harder and more expensive to clean up.

DOD officials contend that because neither the EPA nor any states have ever attempted to use these laws to regulate military training on operational ranges, the exemptions merely codify what are already standard practices. (State and EPA officials disagree with this point, arguing that the amendments actually reverse existing policy under which military munitions may become solid waste after they have been used.) Meanwhile, the DOD adds that it is engaged in a broad voluntary effort to gauge the potential for munitions constituents to migrate from any of its facilities, and that it intends to share the results of this effort with regulators and the public. “The reason DOD is undertaking these assessments is because we realize that our ranges must be operated in a sustainable way,” Willging says. “If they are not, and [migrating contamination] endangers public health, the proposed RRPI provisions will not apply. Therefore, it’s in our best interests to know the condition of our ranges and to respond when contamination threatens to spread.”

Opponents argue that the DOD’s proposals would actually affect both active and closed ranges. “We’ve identified over a dozen DOD operational ranges on the National Priorities List,” says an EPA official speaking off the record. “One could argue that absent an ‘imminent and substantial danger’ finding, EPA would have no jurisdiction under CERCLA to force those cleanups.”

A broad range of critics—including the National Association of Attorneys General and all major environmental organizations—also oppose the proposed CAA extension, arguing that it would extend public exposure to harmful air quality. Moreover, according to a 2004 fact sheet on DOD CAA provisions prepared by staff of the House Committee on Energy and Commerce, there is no evidence to suggest the CAA has ever adversely affected military readiness.

## Culture War?

In late October 2004, the DOD settled its Eagle River Flats lawsuit. As part of the settlement, the agency agreed to a number of key provisions, including—among others—that it obtain a Clean Water Act permit for munitions discharges at the Flats, monitor water quality in the area, promptly clean up munitions that fall outside the immediate impact area, and work with outside experts to study the environmental impacts of bombing.

But the DOD is still committed to its RRPI goals, which it maintains are necessary in order to sustain military readiness. Why is the agency seeking environmental exemptions in the face of such broadly focused opposition? There is no easy answer. Some stakeholders suggest a culture war is at play, pointing out that the DOD has never taken kindly to environmental oversight, believing its national security mission elevates it beyond such concerns. The EPA official says there are many in the DOD itself who don’t support the RRPI’s proposals: “They see it as being driven by operational guys, farther up the chain of command.”

The DOD is currently considering its legislative package for fiscal year 2006. Whether the RRPI will be a part of that package is still being considered in the Pentagon. The next opportunity for DOD officials to present the proposal is likely to emerge when the Congress turns to its next appropriations bill.

## Figures and Tables

**Figure:**
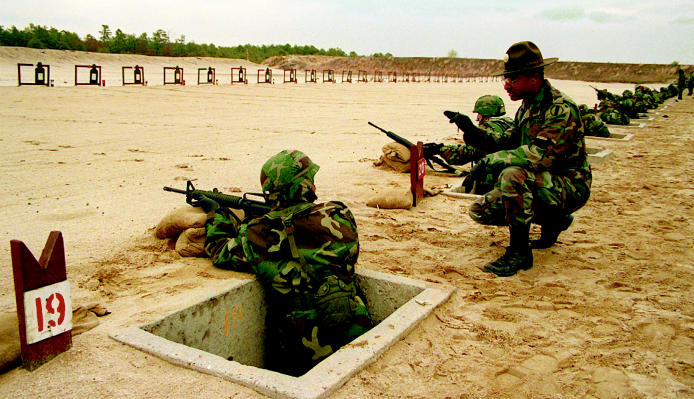
**Catching flak?** The Department of Defense has come under fire for trying to exempt a number of its facilities from environment-protective laws in the name of maintaining optimal military preparedness.

